# Prevalence and associated factors of intimate partner violence against women in Ghana: a secondary analysis of the 2022 Demographic and Health Survey

**DOI:** 10.3389/fpubh.2025.1685386

**Published:** 2025-12-03

**Authors:** Masood Ali Shaikh

**Affiliations:** Department of Medicine, College of Medicine, Korea University, Seoul, Republic of Korea

**Keywords:** intimate partner violence, correlates, human rights, women, Ghana

## Abstract

**Background:**

Intimate partner violence (IPV) is a global public health problem and a violation of human rights that affects nearly half of humanity. It inflicts both physical and emotional suffering, imposing an avoidable and preventable burden on health systems and societies.

**Methods:**

This study used de-identified data from the 2022 Ghana Demographic and Health Survey to conduct a secondary analysis on the lifetime prevalence of IPV and its associated factors. IPV was defined as violence committed by the current or most recent husband or male intimate partner. The analysis involved twelve socio-demographic and related attributes among women aged 15–49 years. Bivariate analysis was performed using simple binary logistic regression models to identify statistically significant factors. These were then used in the multivariable binary logistic regression model to determine their associations with IPV.

**Results:**

Overall, 36.17% of women reported having experienced emotional, physical, and/or sexual violence from a current or most recent husband or intimate partner. The most common form of IPV was emotional violence, affecting 31.32% of respondents, followed by physical violence at affecting 16.85%. In total, 20.62% of women reported to have experienced physical and/or sexual IPV. The educational levels of both the woman and her intimate partner, number of living children, her acceptance of IPV, her partner’s alcohol use, awareness of her father having ever beaten her mother, and the level of marital control exerted by her intimate partner were statistically significant association with IPV in the multivariable logistic regression model.

**Conclusion:**

IPV is associated with multiple complex factors. About 1.8 women out of every five women in Ghana have experienced IPV in their lifetime. These findings emphasize the urgent need for effective public health and economic strategies to reduce this preventable form of violence for women in Ghana.

## Introduction

Intimate Partner Violence (IPV) is a globally pervasive public health problem with serious social, economic, and health implications on women’s health. IPV is defined as “any behavior within an intimate relationship that causes physical, psychological, or sexual harm to those in the relationship” (1). Although IPV affects women of all ages, socioeconomic statuses, and cultural backgrounds, its prevalence and associated factors vary considerably across countries and contexts. Thus, necessitating a country-specific nuanced understanding of its prevalence, associated factors, and consequences. In the World Health Organization’s (WHO) AFRO region, which includes Ghana, the lifetime prevalence of physical and/or sexual IPV for ever-married or partnered women was estimated to be 33% in 2018 (2), compared to the global estimate of 27% (2). A recent United Nations Development Programme (UNDP) report, based on data until 2022, revealed that nearly 90% of people globally hold at least one gender-based bias about women and 25% believe that wife beating is justified (3). This statistic presents a significant challenge to achieving the United Nation’s Sustainable Development Goal (SDG) 5, Target 5.2, which aims to ‘eliminate all forms of violence against all women and girls in the public and private spheres, including trafficking and sexual and other types of exploitation’, by 2030 (4).

Ghana, a West African nation, has made considerable progress in advancing gender equality and women’s empowerment. However, IPV remains a widespread issue affecting women across the country (5–8). Several factors contribute to the perpetuation of IPV in Ghana, including cultural norms that condone violence, patriarchal structures that perpetuate gender inequality, and socio-economic disparities that exacerbate women’s vulnerabilities (9–11).

The Demographic and Health Surveys (DHS), administered by the U.S. Agency for International Development (USAID) through its “The Demographic and Health Surveys Program” (DHS), are often the only nationally and sub-nationally representative data sources for many developing countries. Using standardized methodology, DHS have been carried out in over 90 countries (12). Data from these surveys help reveal the links between IPV and its associated factors. Notably, studies based on DHS and other data sources reveal associations between IPV and critical aspects of women’s lives, including age, education levels of both women and their partners, employment status, family wealth, controlling behaviors exhibited by the male partner, alcohol use by the male partner, and women’s exposure to violence during their upbringing and their perspectives on IPV (13–41). However, these research findings also highlight variations in the magnitude and direction of these associations underscoring the need for studying IPV in each country to better comprehend the nuances of this global public health crisis and women’s’ human rights violation universally. IPV exacts a toll on the victim’s health in terms of poor mental health, anxiety, depression, suicidal thoughts and behavior, unwanted pregnancy, pregnancy loss, as well as post-traumatic stress disorder in children who witness physical IPV at home (42–44).

Despite growing recognition of the urgency to address IPV in Ghana, significant research gaps persist. Limited data availability and methodological challenges hinder efforts to accurately assess the prevalence and dynamics of IPV. Notably, the 2014 Ghana DHS did not include IPV-related questions. Therefore, this study aims to contribute to the limited body of knowledge on IPV in Ghana by analyzing the prevalence and associated factors of IPV using the de-identified data from the most recent, nationally representative 2022 Ghana Demographic and Health Survey that included IPV questions.

## Methods

The framework for this study recognized that IPV is a complex issue shaped by a host of interacting personal, family, and sociocultural factors. Guided by previous research and social learning theory—which suggests that people often repeat behaviors they observe—the study proposed that witnessing parental violence and experiencing intimate partner’s controlling behavior could increase a woman’s risk of IPV. Socioeconomic variables such as employment status, household wealth, and both partners’ education were also considered, since these factors may affect household power dynamics and vulnerability to abuse. Additionally, variables including the number of children, household wealth, women’s acceptance of IPV and her involvement in decision-making were included to capture underlying gender norms and autonomy within relationships, which may further influence the likelihood of IPV occurrence.

### Study area and data source

The 2022 Ghana Demographic and Health Survey (GDHS 2022) was conducted by the Ghana Statistical Service in partnership with the Ministry of Health/Ghana Health Service, with the technical support of ICF International Inc., through the DHS Program. Data were collected from a nationally representative sample of 18,450 households across all 16 regions in Ghana between October 17, 2022 and January 14, 2023.

The DHS program employs a standardized methodology for conducting surveys. For the GDHS 2022, the updated sampling frame was based on the 2021 Population and Housing Census. The selected sample was a stratified sample chosen in two distinct stages from the sampling frame. The first stage involved selecting 618 clusters or Enumeration Areas (EAs) from the sampling frame using a probability proportional to size selection procedure. The second stage involved selecting a fixed number of 30 households in each cluster using equal probability systematic sampling. Based on the WHO guidelines for the ethical collection of domestic violence data, including IPV-related questions, one eligible woman per household was selected and interviewed. This resulted in the successful interview of 5,137 women aged 15–49 years, for the IPV questions. The IPV questions referred to an intimate partner defined as*: “a man with whom a never-married woman is in a relationship that involves physical and/or emotional intimacy and for which the relationship is or has the expectation of being long lasting,”* and a Husband/intimate partner was defined as, “*the current husband for currently married women; the most recent husband for divorced, separated, or widowed women; the current intimate partner for never-married women who currently have an intimate partner; and the most recent intimate partner for never-married women who do not currently have an intimate partner but had one in the past.”* A boyfriend was not considered as an intimate partner if the respondent defined him as “*a man with whom a woman has a casual relationship and who she did not mention as an intimate partner*.”

Comprehensive information about the GDHS 2022 sampling design, methodology, questionnaires, and survey implementation plan can be found in the Ghana DHS 2022 country report, which is available for free download on the DHS program website (12). The Ethical Review Committee of the Ghana Health Service and the ICF International Inc. Institutional Review Board provided ethical clearance for the GDHS 2022. Verbal informed consent was obtained from all participants and/or their legal guardians before the survey was administered. The author did not collect any data. The findings are based on secondary analysis of the de-identified data from the GDHS 2022. Access to these datasets was obtained following successful application, and approval for secondary analysis, from the DHS program.^1^

### Data availability

The findings are based on secondary analysis of the de-identified data from the GDHS 2022. Access to these datasets was obtained after a submission of a brief proposal, and its approval for secondary analysis from the DHS program (see text footnote 1). Anyone can apply and request for access to this data from the DHS program (see text footnote 1) for free. i.e., at no cost.

### Study variables

The GDHS 2022 incorporated a standard domestic violence module derived from a modified Conflict Tactics Scale (CTS), a well-validated and reliable instrument utilized in diverse contexts (45, 46). This module included targeted questions to evaluate emotional, physical, and sexual violence perpetrated by male intimate partners. Details on the computation of outcome and explanatory variables is provided below.

### Outcome variable

IPV was measured as a binary composite variable, coded as 1 if the respondent reported having ever experienced any form of emotional, physical, and/or sexual violence from her current or most recent husband/intimate partner. Specific questions were asked to identify the three types of IPV: emotional, physical, and sexual. Emotional IPV was identified if the respondent reported ever being insulted or made to feel bad about herself, threatened with harm or hurt to herself or to someone she cared about, insulted, or humiliated in front of others, by her current or most recent husband or male intimate partner. Physical IPV was recognized if the respondent replied affirmatively to events such as being pushed, shaken, or objects thrown at, slapped, having arm twisted or hair pulled, punched with a fist or something that could potentially hurt, hit by something harmful, kicked, dragged, beaten, purposefully burned or choked, attacked with a knife, gun, or any weapon by her male intimate partner. Whereas sexual violence was recognized if the respondent reported having been physically forced into unwanted sex, physically forced or forced with threats to perform unwanted sexual acts against her will.

### Explanatory variables

Several studies, based on both DHS data and non-DHS data, have identified the associated factors of IPV. In this secondary analysis of the de-identified DHS data, twelve potential factors associated with IPV were explored, including socioeconomic and demographic characteristics, women’s empowerment and her acceptance of IPV, women’s knowledge of physical IPV at home, use of alcohol by the intimate partner, and his controlling behavior. Socioeconomic factors included household wealth quintile, place of residence (rural or urban), and the number of living children. Assessment of women’s empowerment was determined through the dynamics of her ability to participate, either alone or together with her intimate male partner, in decision-making pertaining to her own healthcare, visiting friends/family, and major purchases. Attitudes towards IPV were examined by evaluating whether the respondents endorsed statements that justified wife beating under specific circumstances, such as refusing sex, arguing, neglecting children, burning food, or leaving home without informing the husband.

The exposure to violence was assessed by determining whether respondents had knowledge of their father ever physically beating their mother. The partner’s controlling behavior was evaluated through confirming experiences such as jealousy or anger when the woman talked with other men, accusations of infidelity, restrictions on social interactions, prohibiting meetings with female friends, and demanding to know her constant whereabouts. Additionally, the study investigated the woman’s intimate partner’s alcohol consumption. Questions regarding the educational attainment of husbands or intimate male partners and women’s participation in decision-making were exclusively directed at women who were either currently married or cohabiting with a male intimate partner.

### Statistical analysis

All analyses were conducted using STATA version 18 (Texas, USA), taking into account the complex survey design. De-identified datasets from the Ghana DHS were obtained for secondary analysis from the DHS website. Descriptive analyses were performed to outline attributes and outcomes. Descriptive statistics included unweighted counts, the number of missing values, and weighted proportions calculated for all variables without imputing missing values. Binary simple logistic regression models were then computed for each explanatory variable to determine the association with the binary IPV outcome. Variables with the statistical significance at the level of *p* < =0.20 in the individual models were retained for the multivariable logistic regression model. Because questions regarding husbands’ or male intimate partners’ educational attainment and women’s participation in decision-making were only posed to women who were either currently married or having a male intimate partner, a second multivariable logistic regression model excluding these factors was conducted, as a sensitivity analysis to determine the impact of the remaining factors on IPV. Multicollinearity among the explanatory variables was assessed using the Variance Inflation Factor (VIF), and the Goodness-of-Fit test was applied to the two final multivariable models to ensure their adequacy. For all binary and the two multivariable models, odds ratios, with 95% confidence intervals and *p*-values were reported. For the two multivariable models, the p-value of less than 5% (*p* < 0.05) was considered statistically significant.

## Results

A total of 5,737 women were selected and interviewed for the domestic violence module. An additional 73 women were selected but could not be interviewed due to lack of privacy, while another 22 women could not be interviewed owing to challenges in contacting them despite multiple attempts. Out of the 5,737 women selected and interviewed for the domestic violence module, the IPV questions were asked from 5,137 women who met the eligibility criteria of either currently or formerly being married or having an intimate partner.

Overall, 36.17% of women surveyed reported having experienced emotional, physical, and/or sexual violence from an intimate partner. Emotional abuse was the most common, affecting 31.32% of respondents, followed by physical violence at 16.85%, and sexual violence at 8.25%. Notably, many women endured multiple forms of intimate partner violence (IPV): 13.60% experienced both physical and emotional violence, 4.51% faced physical and sexual violence, 6.31% encountered emotional and sexual abuse, and 4.17% reported having suffered from all three types of IPV. Overall, 20.62% of women had experienced physical and/or sexual violence from an intimate partner, underscoring the complex nature of IPV. The most frequently reported forms of emotional, physical, and sexual IPV, respectively were: 27.12% of women reported ever being insulted or made to feel bad, 11.58% reported ever being slapped, and 7.37% reported being forced into unwanted sex by the intimate male partner.

Table 1 presents the unweighted counts and weighted proportions for IPV of those affected, as well as the various types of IPV encountered. The analysis considered twelve pertinent factors; however, data on “Husband/intimate partner’s education” and women’s involvement in “Decision making” were not available/missing for 1,378 women. This was because the study specifically targeted these questions to women who were either currently married or had a current intimate partner. Consequently, the results of statistical analyses for these two variables are limited to women who were either currently married or had a current intimate partner.

**Table 1:** Counts and proportions of study variables—Ghana Demographic and Health Survey 2022.

Variable	Unweighted count (*N* = 5,137)	Percentage (weighted)
Outcome variable		
INTIMATE partner violence (emotional, physical, and/or sexual)	Yes = 1,853	36.17 (34.07–38.33)
Emotional violence	Yes = 1,581	31.32 (29.33–33.38)
Physical violence	Yes = 882	16.85 (15.53–18.27)
Sexual violence	Yes = 430	8.25 (7.25–9.37)
Explanatory variables		
Age	15–19y = 332	7.13%
	20–24y = 886	17.72%
	25–29y = 988	17.97%
	30–34y = 985	16.94%
	35–39y = 850	16.30%
	40–44y = 651	13.75%
	45–49y = 445	10.19%
Respondent’s education	No education = 1,280	18.58%
	Primary = 754	14.14%
	Secondary = 2,619	56.69%
	Higher = 484	10.58%
Husband/Partner’s education	No education/don’t know = 1,087	22.35%
	Primary = 366	9.09%
	Secondary = 1,752	53.75%
	Higher = 554	14.81%
	*Not applicable = 1,378	
Occupation	Professional/Technical/Managerial/Clerical/	
	Sales = 876	18.82%
	Not working = 762	14.62%
	Agriculture: self-employed or employee/services/skilled manual/Unskilled manual/other = 3,499	66.56%
Wealth	Poorest = 1,254	16.20%
	Poorer = 1,102	18.04%
	Middle = 1,017	19.98%
	Richer = 969	23.53%
	Richest = 795	22.26%
Residence	Urban = 2,470	56.34%
	Rural = 2,667	43.66%
Children	0 = 902	20.74%
	1–2 = 1,867	35.44%
	3–4 = 1,467	27.19%
	5–11 = 901	16.63%
Decision making	Participated = 3,232	88.70%
	Not participated = 527	11.30%
	*Not applicable = 1,378	
Acceptance	Not justified/don’t know = 3,891	80.42%
	Justified = 1,246	19.58%
Alcohol use by partner/husband	No = 3,831	70.07%
	Yes = 1,306	29.93%
Knowledge of Parental IPV	No/don’t know = 4,499	87.64%
	Yes = 638	12.36%
Controlling behavior	No = 2,018	39.00%
	Yes = 3,119	61.00%

*Questions on ‘Husband/male intimate partner’s education’ and questions pertaining to ‘Decision making’ were asked from only those women who were either currently married or had a current intimate partner.

The respondent profile showed that 40.24% women were aged between 35 and 49 years while the rest were aged betweem 15 and 34; 67.27% had attained secondary or higher education, while 68.56% of women reported similar educational attainment for the partners. Two-quarter (66.56%) were either self employed or were an employee in the agricultural sector/Services/Skilled manual/Unskilled manual/other. About half of the respondents (45.79%) belonged to either the richer or richest wealth quintiles; 56.34% resided in urban areas; 20.74% had no living children. Most women (88.70%) were involved in decision-making and 80.42% expressed acceptance of IPV. Furthermore, 12.36% reported knowing that their father had ever physically beaten their mother, 29.93% reported that their husband/intimate partner consumed alcohol, and 61.00% indicated controlling behavior by their intimate partner/husband.

Table 2 shows the results from both simple and multivariable logistic regression models, highlighting crude and adjusted odds ratios, along with their 95% confidence intervals and statistical significance values. All twelve explanatory variables examined, showed a statistically significant association with the occurrence of intimate partner violence in the simple logistic regression model at the level of <= 0.20. As a result, the multivariable logistic regression model included all twelve covariates. Except for women’s age, her occupation, household wealth, residential status, and participation in decision making, all remaining seven correlates/factors in the final multivariable logistic regression model showed statistically significant associations with women’s experiences of IPV.

**Table 2:** Crude odds ratios and adjusted odds ratios for all statistically significant associations between intimate partner violence and the selected variables – Ghana Demographic and Health Survey 2022.

Explanatory variable	Unadjusted OR	*p*-value	95% CI	Adjusted OR	*p*-value	95% CI
Age						
15–19	Reference			Reference		
20–24	1.45	0.083	0.95–2.21	1.02	0.947	0.50–2.11
25–29	1.40	0.120	0.92–2.15	0.96	0.912	0.46–1.99
30–34	1.63	0.016	1.10–2.42	0.98	0.949	0.47–2.01
35–39	1.42	0.106	0.93–2.16	0.86	0.698	0.39–1.87
40–44	2.37	<0.001	1.55–3.60	1.02	0.959	0.47–2.24
45–49	2.35	<0.001	1.50–3.67	1.11	0.819	0.47–2.62
Education (respondent/women)						
No education	Reference			Reference		
Primary	1.12	0.418	0.85–1.47	1.08	0.681	0.75–1.55
Secondary	0.72	0.002	0.59–0.89	0.92	0.665	0.65–1.32
Higher	0.41	<0.001	0.29–0.57	0.54	0.030	0.31–0.94
Education (husband/partner)						
No education	Reference			Reference		
Primary	1.66	0.008	1.14–2.40	1.66	0.025	1.06–2.58
Secondary	0.75	0.026	0.58–0.97	0.78	0.222	0.53–1.16
Higher	0.49	<0.001	0.36–0.68	0.74	0.239	0.45–1.22
Occupation						
Professional, Technical, Managerial, Clerical, Sales	Reference			Reference		
Not working/don’t know	0.83	0.245	0.61–1.14	1.06	0.770	0.72–1.56
Agriculture: self-employed or employee/household and domestic/services/skilled manual/unskilled manual	1.28	0.030	1.02–1.59	1.07	0.634	0.80–1.45
Wealth						
Poorest	Reference			Reference		
Poorer	1.04	0.764	0.80–1.36	1.07	0.723	0.75–1.53
Middle	0.98	0.888	0.75–1.28	1.33	0.163	0.89–1.99
Richer	0.76	0.029	0.59–0.97	1.13	0.571	0.74–1.73
Richest	0.64	0.002	0.48–0.84	0.97	0.921	0.58–1.63
Residence						
Urban	Reference			Reference		
Rural	1.21	0.042	1.01–1.46	0.94	0.615	0.73–1.21
Children						
No children	Reference			Reference		
1–2 children	1.39	0.012	1.08–1.81	1.34	0.167	0.88–2.04
3–4 children	1.72	<0.001	1.32–2.22	1.51	0.077	0.96–2.37
5–11 children	2.48	<0.001	1.83–3.36	1.82	0.029	1.06–3.11
Decision making						
Did not participate	Reference			Reference		
Participated	1.24	0.140	0.93–1.65	1.11	0.485	0.82–1.50
Acceptance of IPV						
Not justified	Reference			Reference		
Justified	1.76	<0.001	1.46–2.14	1.41	0.003	1.13–1.76
Alcohol use by partner/husband						
Does not use alcohol	Reference			Reference		
Uses alcohol	2.68	<0.001	2.22–3.24	2.38	<0.001	1.90–2.99
Knowledge of parental IPV						
No	Reference			Reference		
Yes	2.14	<0.001	1.66–2.75	1.73	0.001	1.25–2.39
Controlling behavior						
No	Reference			Reference		
Yes	3.54	<0.001	2.92–4.30	4.20	<0.001	3.35–5.27

OR, Odds Ratio; CI, Confidence Interval.

The multivariable logistic regression model shown in Table 2 displays seven statistically significant associations related to IPV factors. Compared with women with no education, higher education in women bestowed protection from IPV (aOR: 0.54; 95% CI: 0.31–0.94); conversely, compared with no education, women whose partner had primary education were more likely to report IPV (aOR: 1.66; 95% CI: 1.06–2.58). Women with five or more living children were more likely to report IPV versus those with no children (aOR: 1.82; 95% CI: 1.06–3.11); Acceptance of IPV was associated with a higher odds of reporting IPV compared to non-acceptance (aOR:1.41; 95% CI: 1.13–1.76). The remaining three factors, i.e., alcohol use by the partner (aOR: 2.38; 95% CI: 1.90–2.99), knowing about mother being beaten by father (aOR: 1.73; 95% CI: 1.25–2.39), and controlling behavior exercised by the partner (aOR: 4.20; 95% CI: 3.35–5.27), were all statistically highly significantly associated with IPV.

Table 3 compares the outcomes from two separate multivariable logistic regression models, presented side by side, with one model including husband/male intimate partners’ educational attainment and women’s involvement in decision making replicated from Table 2, and the other model excluding them. There were a total of 1,378 women who were either not currently married or not currently having a male intimate partner. In the first model, which included the correlates of husband/male intimate partners’ educational attainment and decision-making involvement of women; data for these 1,378 women were omitted (row-wise deleted), leaving a sample of those women who met the criteria of being currently married or having a current intimate partner. The second model, which excluded these two correlates, including the 1,378 women who were either not currently married or not having an intimate partner, but had been in intimate relationship in the past.

**Table 3:** Comparative analysis of intimate partner violence correlates: models with and without husband/partner’s ‘educational attainment’ correlate—Ghana Demographic and Health Survey 2022.

Explanatory variable	Model with Husband/Partner’s education and Women’s involvement in decision making			Model without Husband/Partner’s education and Women’s involvement in decision making		
	Adjusted OR	*p*-value	95% CI	Adjusted OR	*p*-value	95% CI
Age						
15–19	Reference					
20–24	1.02	0.947	0.50–2.11	1.36	0.183	0.87–2.13
25–29	0.96	0.912	0.46–1.99	1.18	0.522	0.71–1.96
30–34	0.98	0.949	0.47–2.01	1.25	0.350	0.78–2.00
35–39	0.86	0.698	0.39–1.87	0.98	0.921	0.59–1.61
40–44	1.02	0.959	0.47–2.24	1.48	0.143	0.88–2.48
45–49	1.11	0.819	0.47–2.62	1.52	0.160	0.85–2.73
Education (respondent/women)						
No Education	Reference					
Primary	1.08	0.681	0.75–1.55	1.10	0.539	0.81–1.49
Secondary	0.92	0.665	0.65–1.32	0.89	0.392	0.69–1.16
Higher	0.54	0.030	0.31–0.94	0.64	0.034	0.42–0.97
Education (husband/partner)						
No Education	Reference					
Primary	1.66	0.025	1.06–2.58	Excluded		
Secondary	0.78	0.222	0.53–1.16			
Higher	0.74	0.239	0.45–1.22			
Occupation						
Professional, Technical, Managerial, Clerical, Sales	Reference			Reference		
Not working/don’t know	1.06	0.770	0.72–1.56	0.84	0.293	0.60–1.17
Agriculture: self-employed or employee/household and Domestic/Services/Skilled manual/Unskilled manual	1.07	0.634	0.80–1.45	1.00	0.986	0.78–1.28
Wealth						
Poorest	Reference			Reference		
Poorer	1.07	0.723	0.75–1.53	1.10	0.546	0.81–1.49
Middle	1.33	0.163	0.89–1.99	1.09	0.607	0.79–1.50
Richer	1.13	0.571	0.74–1.73	0.89	0.514	0.64–1.25
Richest	0.97	0.921	0.58–1.63	0.90	0.605	0.60–1.34
Residence						
Urban	Reference			Reference		
Rural	0.94	0.615	0.73–1.21	1.01	0.913	0.81–1.26
Children						
No children	Reference			Reference		
1–2 children	1.34	0.167	0.88–2.04	1.39	**0.037**	1.02–1.89
3–4 children	1.51	0.077	0.96–2.37	1.76	**0.002**	1.24–2.49
5–11 children	1.82	0.029	1.06–3.11	2.23	<0.001	1.45–3.44
Decision making						
Did not participate	Reference			Excluded		
Participated	1.11	0.485	0.82–1.50			
Acceptance of IPV						
Not justified	Reference			Reference		
Justified	1.41	0.003	1.13–1.76	1.39	0.001	1.14–1.70
Alcohol use by partner/husband						
Does not use alcohol	Reference			Reference		
Uses alcohol	2.38	<0.001	1.90–2.99	2.38	<0.001	1.95–2.91
Knowledge of parental IPV						
No	Reference			Reference		
Yes	1.73	0.001	1.25–2.39	1.80	<0.001	1.35–2.39
Controlling behavior						
No	Reference			Reference		
Yes	4.20	<0.001	3.35–5.27	3.84	<0.001	3.14–4.69

OR, Odds Ratio; CI, Confidence Interval.

*P-values in bold indicate categories of the variable ‘Children’ whose statistical significance differed between the two multivariable models.

The results from the two multivariable logistic regression models as given in Table 3 demonstrate consistent directions of association for all statistically significant correlates. However, two categories of the correlate ‘number of living children’ that were not statistically significant in the first model (that included husband/male intimate partner’s education and women’s involvement in decision-making) were found to be statistically significant in the second model that excluded them. These differences in *p*-values are indicated in bold. In the first model, no statistically significant association was found for the categories of having 1–2 children or 3–4 children, compared with no children; only having 5 or more children was significantly associated with IPV. However, in the second model, all three categories of the number of children showed a statistically significant association with IPV. For the correlates of women’s education, partner’s alcohol use, awareness of the father’s physical IPV against the mother, acceptance of IPV, and controlling behavior by the partner, the adjusted odds ratios remained relatively consistent.

VIF results were less than 2.37 for all covariates; the results of the goodness-of-fit test indicated that both multivariable logistic regression models for the IPV were satisfactory [*F*(9,578) = 1.12; *p*-value: 0.3492] for the model that included husband/male intimate partner’s educational attainment and women’s involvement in decision making. Whereas for the multivariable model that excluded these two correlates, statistics were [*F*(9,578) = 1.21; *p*-value: 0.2865].

## Discussion

Evidence adduced globally indicates that IPV against women is endemic and no country is immune to it. Women constitute half of humanity, and their health is an important determinant of social development. Although intimacy and violence might seem incompatible, they unfortunately coexist as a widespread concern globally; imposing substantial burden for societies, affected families, and health systems. In Ghana, IPV continues to be a major concern, profoundly affecting the lives of many individuals. In this study, based on the secondary analysis of the most recent nationally representative DHS conducted in Ghana, the prevalence and associated factors of IPV were estimated. The GDHS 2022 findings revealed that 36.17% of ever-partnered women aged 15–49 in Ghana had experienced physical, sexual, and/or emotional IPV from their current or most recent husband/intimate partner at some point in their lives; which translates to about 1.8 women out of every five women. In comparison, the global lifetime prevalence rate for physical and/or sexual IPV was 27% in 2018, while the WHO’s AFRO region had a prevalence rate of 33% (2). In contrast, Ghana’s lifetime prevalence of physical and/or sexual IPV was 20.62%.

Although DHS have been conducted in several countries around the world using standard methodologies and tools allowing for internationally comparable estimates of IPV, the substantial heterogeneity across countries underscores the fact that ultimately “all epidemiology is local” (47). Hence, a better appreciation of nuanced differences in the profile and the associated factors of IPV is essential for each country to tailor their policies and interventions to be aligned with the United Nations Sustainable Development Goals.

Emotional IPV was the most commonly reported form of violence among Ghanaian women, consistent with findings from other countries using DHS data (34, 35). This contrasts with several other countries where physical violence is reported as the predominant form (13, 20, 22, 23). In the multivariable model, the respondent’s age, occupation, household wealth, residency status, and women’s involvement in decision-making were not statistically significant. The multivariable model results for IPV factors in Ghana, contrasting with other countries, present a kaleidoscope of victim profiles. Numerous studies have shown that greater household wealth and economic status confer protection from IPV (14, 16, 20, 28, 33), although some studies have found no association between wealth and IPV (22, 23, 34, 35). Studies indicate that women’s income and unemployment status can act as protective factors against IPV (36, 41). However, if a woman earns more than her partner, it may also be considered a risk factor (16, 41). Additionally, several studies indicate that occupational status does not appear to be associated with IPV (20, 22, 23, 34, 35). Likewise, the relationship between residential status, whether urban or rural, and IPV is complex. Studies have shown conflicting results, with some reporting higher IPV rates in rural areas (25, 28), other finding reporting lower IPV rates (17), and some observing no statistically significant association between residency and IPV (20, 22, 23, 34, 35). Women’s age was another factor that was not found to be statistically significant in the multivariable model. The relationship between age and IPV has been reported in previous studies with mixed findings indicating that older age is linked to higher IPV (13, 22), while other studies suggest that older age offers some protection against IPV (14, 38). Similarly, younger age has been associated with increased IPV (15), but there are also reports of no significant link between age and IPV (20, 23, 34, 35). Older women might be more likely to experience IPV because of their exposure duration to intimate relationships and unions; while husbands/intimate partners of younger women, ostensibly younger themselves, might lack skills in handling relationship stresses.

Finally, participation in decision-making by women was also not statistically significantly associated with IPV. Such participation confers some measure of autonomy and empowerment in daily life, and enhances relationship quality. While some studies have reported Lower IPV odds with such participation (21), others have found no statistically significant association (20, 34). However, in this study, with 88.70% of respondents involved in decision-making, this factor probably lost its discriminatory power in the multivariable model. This range of conflicting associations highlights the intricate social, cultural, and demographic factors influencing the association between age and IPV.

The multivariable model identified seven statistically significant associations with IPV: number of living children, acceptance of IPV by women, educational level of women and their husbands/intimate partners, having knowledge of father ever physically beating mother, controlling behavior demonstrated by the intimate partner, and his use of alcohol. Among these, the association with women’s knowledge of father having ever beaten mother, partners’ controlling behavior, and his use of alcohol have been consistently reported, in terms of their strong positive associations with IPV. While for the remaining factors, the IPV’s association is rather intricate and complex.

Compared to women without children, having more children has been a rather consistent factor in previous studies (15, 17, 22, 23, 28, 34–36). However, one study found no association between the number of living children and IPV (20), while another reported higher IPV rates among infertile women (41). These findings suggest that in patriarchal societies, men might resort to violence when they struggle with their traditional provider role. Similarly, infertile women may face violence due to their inability to procreate, reflecting societal expectations of their child-bearing role. Studies have shown equivocal results regarding the acceptance of IPV by women; some studies report a positive link between acceptance and IPV (13, 17, 22, 23, 35, 40), while others report no association (20, 34). Women’s acceptance of IPV is a learned behavior shaped by cultural norms, practices, personal upbringing, and experiences. This acceptance can perpetuate a self-fulfilling cycle within intimate relationships.

Education equates to enlightenment and encourages resorting to peaceful means of navigating differences and life’s difficulties. The association between educational attainment of women and her partner with IPV showed a paradoxical relationship. In contrast to women with no education, those with higher education had 46% lower odds of experiencing IPV. While compared to women whose partners had no education, those with primary education reported an adjusted odds ratio of 1.66, indicating that their odds of reporting IPV were 66% higher. This may seem counterintuitive, but it reflects the complex factors driving IPV in Ghana. Low educational level in women has been associated with higher IPV rates (15, 25), while primary and higher educational levels have been linked to lower IPV (13, 16, 20, 35). However, some studies have found no association between these factors (22, 23, 34).

Similarly, several studies indicate that low educational attainment in male partners is associated with higher IPV reporting by women (15, 21, 36), whereas primary or higher education levels in male partners correlate with lower IPV (13, 16, 20, 35). One study, however, found that women whose partners had secondary-level education experienced higher IPV compared to those with uneducated partners (22). There are also reports of no association between IPV and a partner’s educational attainment (34). However, it’s essential to recognize that sociocultural contexts can sometimes overshadow the protective effects of educational attainment, impacting women’s rights and safety within intimate relationships.

Being aware that a father has perpetrated physical IPV against the mother has consistently been associated with higher levels of IPV in women (14, 17, 20, 22, 24, 26, 31, 34, 35). Recognizing that one’s father has engaged in physical IPV against one’s mother might inadvertently create an appearance of normalcy for such violence within intimate relationships and contribute to the perpetuation of IPV in the relationships of daughters. The link between increased IPV and controlling behavior exhibited by male partners is also well-established (14, 17, 20, 22, 27, 34, 35). It seems likely that controlling behavior can escalate into IPV. Lastly, the association between high IPV and a partner’s alcohol consumption is extensively documented (14, 15, 17–19, 21–23, 29, 34–36). Alcohol’s impact on reducing inhibitions and increasing the likelihood of violent behavior is well established.

In bivariate analysis, the factors of educational attainment of husbands/intimate partners and women’s involvement in decision-making, both met the inclusion criteria for multivariable model. These two covariates were collected only from those women who were either currently married or in an intimate relationship. Among the total sample of 5,137 women, 1,378 did not meet these criteria and were therefore not asked about these two questions. Consequently, a second multivariable model was developed, excluding the correlate of husband/male intimate partner’s educational attainment and women’s involvement in decision-making, to include all available records (i.e., women who were ever in an intimate relationship) and assess its impact on the findings.

The outcomes of the second multivariable model confirmed the results observed in the first model. However, one exception was the number of living children: in the first multivariable model (inclusive of educational attainment of husbands/male intimate partners and women’s involvement in decision making), only having 5 or more children was statistically significant and associated with IPV, compared with having no children. In contrast, the second multivariable model (which excluded the covariates of educational attainment of husbands/male intimate partners and women’s involvement in decision making) showed that compared with women with no children, those who had one to two children, three to four children, and five or more children were all statistically significant and associated with IPV. Additionally, there was a clear gradient in the adjusted odds ratios, with having more children being associated with higher odds of IPV. Although this gradient was also present in the first multivariable model, it did not reach statistical significance. These results suggest that perhaps having children may contribute to financial pressures on male intimate partners, resulting in IPV. Alternatively, it could be due to the fact that women with more children tend to be older and thus have a longer ‘exposure’ time to experience IPV. The other discernible difference between the two models was in the controlling behavior exhibited by male intimate partners. In the first model, the adjusted odds of controlling behavior associated with IPV were 4.22, whereas in the second model, it was 3.84. Therefore, the adjusted odds were lower in the second model. This finding is difficult to theorize about, but it might suggest that some women leave intimate relationships at the very first warning signs of controlling behavior before it escalates to full-blown IPV.

While this study has notable strengths, such as utilizing data from the recent and only nationally representative survey investigating IPV, it also has limitations. Focus on heterosexual relationships eliminates those women who experienced IPV in same-sex relationships, possibly underestimating the overall burden of IPV in Ghana. Additionally, cross-sectional surveys like the DHS cannot establish causality or ‘reverse causality’ due to their inability to establish a clear order of preceding and following events. As past IPV experiences may shape attitudes toward IPV or may affect recall of parental violence. Secondly, the self-reported nature of data collection in DHS might also make them susceptible to underestimation, owing to perceived shame and stigma felt by respondents.

Identifying and understanding both individual attributes and societal norms is critical for effective IPV control interventions. The findings gleaned in this study can inform more effective planning and implementation of such strategies in Ghana.

## Conclusion

More than one in three women in Ghana reported experiencing at least one form of IPV – emotional, physical, or sexual – perpetrated by their current or most recent partner requires solutions that are grounded in the country’s sociocultural realities. Results point to the strong influence of intergenerational cycle of violence, with women who knew that their fathers had physically beaten their mothers were far more likely to report IPV themselves. The IPV risk was heightened among those with controlling partners and those whose partners used alcohol. Women’s acceptance of IPV as justified was another key correlate. Lower education levels and having more children increased her likelihood of experiencing IPV. These findings suggest clear priorities for Ghana. Public health campaigns need to challenge and reduce the normalization of violence within families. Expanding women’s access to education and income opportunities can reduce economic dependence and IPV risk. And importantly, prevention efforts must include men—promoting equitable gender attitudes and addressing controlling behaviors and alcohol misuse at their source.

## Data Availability

The data analyzed in this study is subject to the following licenses/restrictions: the findings provided here are based on secondary analysis of the deidentified data from the Ghana Demographic and Health Survey (GDHS) of 2022. Access to these datasets was obtained after a submission of a brief proposal, and its approval for secondary analysis from the DHS program (https://dhsprogram.com/). Anyone can apply and request for access to this data from the DHS program (https://dhsprogram.com/) for free i.e., at no cost. Requests to access these datasets should be directed to the DHS program (https://dhsprogram.com/) for free i.e., at no cost.

## Footnotes

1
https://dhsprogram.com/
